# Comparison of Hemodynamic Response to Laryngoscopy Using Miller and McCoy Blade

**DOI:** 10.7759/cureus.24914

**Published:** 2022-05-11

**Authors:** Thejeswini Mahadevaiah, Deepak T S, Roopa Rani, Vikas K N, Shwetha G M

**Affiliations:** 1 Department of Anaesthesiology, Mathikere Sampangi Ramaiah Medical College, Bangalore, IND; 2 Department of Emergency Medicine, Mathikere Sampangi Ramaiah Medical College, Bangalore, IND; 3 Department of Anaesthesiology, North Bangalore Hospital, Bangalore, IND

**Keywords:** pressor response, mccoy blade, miller blade, intubation, laryngoscopy

## Abstract

Background: The most commonly used equipment to aid endotracheal intubation is a laryngoscope, and the procedure performed is known as laryngoscopy, which leads to profound cardiovascular effects. The process of laryngoscopy causes the release of catecholamines, thereby leading to marked pressor responses and tachycardia. The process of laryngoscopy can be made easier by the use of various types of laryngoscopic blades. The McCoy blade is a modification of the standard Macintosh blade that incorporates a hinged tip blade. It allows elevation of the epiglottis while decreasing overall laryngeal movement. A Miller blade is a straight blade with a slight upward curve near the tip. It is found that the force exerted, head extension, and cervical spine movement are less with the Miller blade. This study was undertaken to compare changes in haemodynamic parameters before, during, and after laryngoscopy using these two blades.

Materials and methods: Following institutional ethical committee approval and obtaining informed written consent, 100 patients of American Society of Anesthesiologists (ASA) grades I and II in the age group of 18-45 years of either sex undergoing elective surgeries under general anaesthesia were included in the study. The patients were randomly allocated into two groups of 50 patients each. Group Mc - laryngoscopy was performed using a no. 3 McCoy blade. Group Ml - laryngoscopy was performed using a no. 2 Miller blade. The laryngoscopic view was compared using Cormack and Lehane grading. Haemodynamic parameters before, during, and after laryngoscopy were recorded.

Results: Hemodynamic parameters including heart rate (HR), systolic blood pressure (SBP), diastolic blood pressure (DBP), and mean arterial pressure (MAP) were increased in both the groups but were statistically and clinically significant in the Miller group with p≤0.001.

Conclusion: McCoy blade is associated with a significantly more stable hemodynamic response to laryngoscopy in comparison with the Miller blade.

## Introduction

Endotracheal intubation has become an integral part of balanced anaesthesia since its introduction by Rowbotham and Magill in 1921 because of its ability to secure the airway and also isolate the airway. Cardiovascular responses to endotracheal intubation are well documented [1,2].

Laryngoscopes are the most widely used equipment for inserting endotracheal tubes into the trachea. Moreover, it aids in the proper visualisation of laryngeal structures. Many varieties of laryngoscopes are available, ranging from rigid to flexible fiberoptic devices [3]. The fundamental aim of laryngoscopy is to obtain optimum visualisation of laryngeal structures and vocal cords, thereby leading to successful and smooth endotracheal intubation. The success of laryngoscopy lies in the proper alignment of the oral, pharyngeal, and laryngeal axis together with an extension of the head and flexion of the neck at the atlantooccipital joint and the lower cervical spine, respectively [4]. Laryngoscopy leads to the release of catecholamines (norepinephrine from adrenergic nerve terminals and secretion of epinephrine from the adrenal medulla), during manipulation of the airway thereby causing tachycardia and hypertension, mediated by sympathetic chain ganglia and cardio accelerator fibres [4].

Direct laryngoscopy and endotracheal intubation are noxious stimuli that can lead to adverse responses in the physiologic systems, including cardiovascular and respiratory. In case, endotracheal intubation is performed under a light plane of anaesthesia, then it leads to significant tachycardia and hypertension. The greater the lifting force of the laryngoscope, the greater the magnitude of the cardiovascular responses. After five seconds of direct laryngoscopy, the arterial blood pressure begins to rise, peaking in the next one to two minutes and finally returning to baseline levels within five minutes. In patients with underlying cardiac disease, such haemodynamic changes can result in myocardial ischaemia/infarction, but fortunately, it causes little harm to healthy patients [5].

Successful endotracheal intubation and maintaining a patent airway carry a pivotal role in providing adequate oxygenation and ventilation. Failure to do so for even a brief period can endanger the life of the patient. Inadequate ventilation, accidental oesophageal intubation, and difficult tracheal intubation are the three main causes of airway/respiratory-related injuries [6]. Therefore, the blades used for laryngoscopy and endotracheal intubation should trigger a minimal stress response and simultaneously facilitate an optimum laryngoscopic view for successful endotracheal intubation [7-9]. In this study, we aim to evaluate the alterations in haemodynamic parameters before, during, and after laryngoscopy using Miller and McCoy blades.

## Materials and methods

In the study, 100 adult patients (American Society of Anesthesiologists [ASA] grade I/II) who were posted for abdominal or gynaecological procedures at MS Ramaiah Medical College and Research Institute, Bangalore, were enrolled during the period from November 2018 to May 2020. Institutional ethical committee approval via letter no. SS-1/EC/31/2018 was obtained. Inclusion criteria comprised adult patients (between the age of 18 and 45 years) with ASA grade I/II undergoing elective surgery under general anaesthesia requiring tracheal intubation with informed consent. However, the patients with anticipated or unanticipated difficult intubation, who did not give consent to participate in the study, had a history of chronic obstructive pulmonary disease/asthma, cardiac disease, any co-morbidity, allergic to any of the drugs used in the study, whose weight is more than 20% of ideal body weight were excluded from the study. After computer-generated randomisation, all patients were equally divided into two groups: group Mc - laryngoscopy performed using a no. 3 McCoy blade and group Ml - laryngoscopy performed using a no. 2 Miller blade.

All the patients were visited the day before surgery and pre-anaesthetic counselling was done. Airway assessment was done using Mallampati grading and thyromental distance. All the patients received Tab. Alprazolam 0.5 mg orally on the night before surgery and Tab. Ranitidine 150 mg on the night and two hours before the surgery. On the day of surgery, the intravenous line was secured using an 18-gauge cannula after arrival in the anaesthetic room, and basal parameters (pulse rate, systolic/diastolic blood pressure [SBP/DBP], oxygen saturation, temperature) were recorded. Injection Fentanyl 2 µg/kg was injected ten minutes before induction, and parameters were again recorded after this. Intraoperative monitoring was performed with pulse oximetry, automated non-invasive blood pressure measurement, electrocardiography, and end-tidal CO_2_ concentration.

After preoxygenation of the patients with 100% oxygen for three minutes, the patients were induced with Inj. Thiopentone 5 mg/kg and relaxed with Inj. Vecuronium 0.08 mg/kg. The patient was mask ventilated using a face mask with 50% oxygen and 50% nitrous oxide through the Bain breathing circuit.

After three minutes of giving Inj. Vecuronium and confirming the intensity of block using neuromuscular monitoring, once the profound block is achieved (no response to Train-of-four stimulation and post-tetanic count = 1), laryngoscopy is performed either by using Miller (group Ml) or McCoy (group Mc) enabling a clear view of the vocal cords.

Laryngoscopy by using either of the blades is performed in the sniffing position. While performing laryngoscopy with the McCoy blade, the tip lies in the vallecula and the epiglottis is lifted indirectly. While performing with the Miller blade, the tip is posterior to the epiglottis and it is lifted directly.

Laryngoscopy with tracheal intubation is performed within 15 seconds. Bilateral air entry was checked clinically and by capnography, and the tube was secured. The surgical procedure, including skin incision, is delayed for the first 15 minutes after intubation. Haemodynamic parameters are recorded after induction and then after laryngoscopy starting at one minute and recorded at two-minute intervals for the first 15 minutes. The hemodynamic variation <20% from baseline was considered normal.

Data were compiled, and continuous data were presented as mean and standard deviation (SD), while categorical data were presented as percentages. Data analyses were done using the chi-square test for qualitative comparison. Paired and unpaired t-tests were used for quantitative parameters. P<0.05 was considered statistically significant.

## Results

Of the 100 patients enrolled in the study, all successfully participated in the study, and no drop-outs were found. The mean age in the Miller and McCoy groups was 31.88 ± 7.56 and 31.72 8.20, respectively (p=0.919). On comparing the mean weight between the two groups, no significant difference was observed (p=0.587). In the Miller group, the mean height was 158.76±9.73, whereas in the McCoy group, it was 161.27±7.72 (p=0.23) (Table 1).

**Table 1:** Demographic distribution of patients

Parameters	Miller group (mean ± SD)	McCoy group (mean ± SD)	P-value
Age (years)	31.88 ± 7.56	31.72 ± 8.20	0.919
Weight (kg)	55.96 ± 6.512	56.70 ± 7.063	0.587
Height (cm)	158.76 ± 9.73	161.27 ± 7.72	0.23

Table 2 depicts that pre-induction heart rate (HR) and post-induction heart rate were comparable in both groups. But it was statistically and clinically higher in the Miller group compared to the McCoy group after laryngoscopy and one minute after laryngoscopy. After three min of laryngoscopy again, the heart rate remained comparable in both groups.

**Table 2:** Comparison of heart rate (beats/min) in two groups of patients *p<0.05

Heart rate (beats/min)	Miller group (mean ± SD)	McCoygroup (mean ± SD)	t-value	p-value
Pre-induction	80.64 ± 5.45	79.22 ± 6.71	1.161	0.249
Post-induction	83.66 ± 4.91	81.76 ± 6.27	1.686	0.095
After laryngoscopy	109.7 ± 8.99	102.76 ± 8.29	4.009	<0.001*
1 min	116.06 ± 9.57	109.94 ± 8.47	3.384	0.001
3 min	108.36 ± 12.28	106.56 ± 8.92	0.838	0.404
5 min	99.42 ± 12.92	98.7 ± 7.47	0.341	0.734
7 min	91.98 ± 8.57	92.08 ± 6.12	0.067	0.947
9 min	88.82 ± 7.52	87.62 ± 5.84	0.89	0.375
11 min	84.96 ± 7.52	82.92 ± 5.16	1.581	0.117
13 min	81.56 ± 5.10	79.96 ± 5.37	1.527	0.130
15 min	79.32 ± 3.91	78.24 ± 4.60	1.263	0.21

Table 3 depicts that pre-induction and post-induction SBP were comparable in both groups. The rise in SBP started after laryngoscopy and was statically and clinically higher in the Miller group compared to the McCoy group after laryngoscopy and one minute after laryngoscopy. Following three minutes, the values remained comparable in both groups again.

**Table 3:** Comparison of systolic blood pressure in two groups of patients *p<0.05

SBP (in mmHg)	Miller Group	McCoy Group	t-value	p-value
Pre-induction	130.82 ± 11.21	131.78 ± 11.47	0.423	0.673
Post-induction	129.02 ± 11.89	131.04 ± 11.43	0.865	0.389
After laryngoscopy	152.54 ± 10.81	146.18 ± 13.93	2.550	0.012*
1 min	160.16 ± 11.42	152.06 ± 13.72	3.207	0.002*
3 min	154.2 ± 12.95	151.58 ± 14.20	0.963	0.338
5 min	143.58 ± 12.56	141.14 ± 11.44	1.015	0.313
7 min	133.56 ± 10.94	133.82 ± 8.9	0.13	0.897
9 min	127.46 ± 10.82	130.72 ± 7.95	1.71	0.089
11 min	123.50 ± 10.89	126.50 ± 6.68	1.65	0.100
13 min	120.96 ± 9.93	124.06 ± 9.44	1.6	0.113
15 min	119.68 ± 10.50	121.78 ± 8.81	1.083	0.282

Table 4 depicts that DBP values were comparable in both the groups during pre-induction and post-induction. It was found to be statistically and clinically higher in the Miller group compared to the McCoy group after laryngoscopy and one minute later. The values were comparable in both groups three minutes after laryngoscopy.

**Table 4:** Comparison of diastolic blood pressure in two groups of patients *p<0.05

DBP (mmHg)	Miller group	McCoy group	t-value	p-value
Pre-induction	76.38 ± 6.22	76.72 ± 5.76	0.283	0.778
Post-induction	74.12 ± 6.46	75.74 ± 5.36	1.364	0.176
After laryngoscopy	93.62 ± 4.81	85.54 ± 5.74	7.621	<0.001*
1 min	98.30 ± 5.16	90.74 ± 5.84	6.855	<0.001*
3 min	88.90 ± 5.04	88.30 ± 5.40	0.574	0.568
5 min	84.04 ± 5.78	84.20 ± 4.46	0.155	0.877
7 min	81.84 ± 8.06	79.22 ± 5.71	1.874	0.064
9 min	77.92 ± 8.10	77.22 ± 6.27	0.483	0.630
11 min	74.58 ± 9.48	75.48 ± 6.95	0.541	0.590
13 min	72.56 ± 10.64	73.08 ± 5.01	0.313	0.755
15 min	70.84 ± 10.72	72.00 ± 5.03	0.692	0.491

Table 5 depicts mean arterial pressure (MAP) values that were comparable in both the groups recorded during pre-induction and post-induction. The values were found to be statistically and clinically higher in the Miller group compared to the McCoy group, recorded after laryngoscopy and one minute following laryngoscopy. However, the values remained comparable after three minutes in both groups.

**Table 5:** Comparison of mean arterial pressure in two groups of patients *p<0.05

DBP (mmHg)	Miller group	McCoy group	t-value	p-value
Pre-induction	94.50 ± 6.99	95.34 ± 6.41	0.626	0.533
Post-induction	92.46 ± 7.04	94.18 ± 6.05	1.31	0.193
After laryngoscopy	113.26 ± 5.66	105.75 ± 7.41	5.688	<0.001*
1 min	118.92 ± 6.03	111.38 ± 7.25	5.652	<0.001*
3 min	111.02 ± 6.05	109.36 ± 7.39	1.228	0.222
5 min	103.88 ± 6.68	103.22 ± 5.53	0.538	0.592
7 min	99.10 ± 7.73	97.42 ± 5.36	1.262	0.210
9 min	94.42 ± 7.97	95.12 ± 5.28	0.518	0.606
11 min	90.60 ± 9.09	92.86 ± 5.58	1.498	0.137
13 min	88.72 ± 9.21	89.84 ± 5.57	0.735	0.464
15 min	87.08 ± 9.80	88.64 ± 5.03	1.001	0.319

## Discussion

The cornerstone of successful endotracheal intubation and general anaesthesia is smooth and good laryngoscopy. So far, various types of laryngoscope blades have been designed and used for endotracheal intubation. The proper visualisation of the larynx and adjacent structures depends on the shape and design of the laryngoscope blade. Not only is the view of laryngeal structures important, but the blade also marks a key element in inducing a trigger of the major stress response in the form of the release of catecholamines, leading to haemodynamic responses in the form of tachycardia and hypertension, which may prove detrimental to patients with known cardiovascular disease [9].

The cornerstone of successful laryngoscopy and intubation lies in the pre-operative prediction of difficult airways and thereby permitting anesthesiologists to take precautions to decrease the risk [5]. It is important to adopt multiple approaches in the prediction of the difficult airway, as each test for airway examination may predict a separate aspect. In the present research, we adopted the same approach to airway prediction in either of the groups. Since none of the prediction parameters guarantees perfect airway assessment during laryngoscopy and intubation, innovations in the field of different types of blades are also required. The demand for easy laryngoscopy together with stable haemodynamics is the need for this type of study.

The haemodynamic response in the Miller group to laryngoscopy, shown by a rise in HR (26.92%), SBP (16.91%), DBP (23.10%), MAP (20.21%) above baseline values, and that in the McCoy group, shown by a rise in HR (22.95%), SBP (10.99%), DBP (11.82%), MAP (11.23%) above baseline values, were compared. This revealed that haemodynamic response to laryngoscopy was less in the McCoy group compared to the Miller group with p<0.001, as supported by Nishiyama et al. in their study [10].

Nishiyama et al. compared the stress response to laryngoscopy without endotracheal intubation using three different laryngoscope blades: Miller, Macintosh, and McCoy. Haemodynamic parameters in the form of blood pressure and heart rate were observed in 58 patients, together with plasma concentration of catecholamines in 29 patients before, during, and after laryngoscopy [10]. They observed that the stress response to laryngoscopy without intubation was least with the McCoy blade whereas highest with the Miller blade [10].

In our study, we compared the stress response to laryngoscopy using haemodynamic parameters, and no biochemical parameters were used. We compared the values for the first 15 minutes after laryngoscopy. The HR, SBP, DBP, and MAP values were statistically and clinically higher in the Miller group compared to the McCoy group after laryngoscopy and one minute after laryngoscopy. The values remained comparable in both groups afterward.

McCoy et al. conducted a study on 20 patients where they compared the stress response to laryngoscopy using the McCoy and Macintosh blades. The haemodynamic parameters together with catecholamine concentrations were compared in all patients before and after laryngoscopy. They observed that there was a significant increase in both arterial blood pressure by 27% and heart rate by 33% after laryngoscopy using the Macintosh blade (p<0.01). The McCoy blade carried an advantage in that no significant change in either of the above-mentioned hemodynamics was observed. They finally came up with the conclusion that the stress response to laryngoscopy is less marked with the McCoy blade, maybe due to the less lifting force imparted during the laryngoscopy and finally obtaining a better view of the laryngeal structures [11].

Haidry and Khan compared the hemodynamic response to tracheal intubation with Macintosh and McCoy laryngoscopes. In their study, the maximum change in heart rate and systolic blood pressure was 18.7% and 22.9% in the Macintosh group, respectively, whereas the fluctuation in heart rate and systolic blood pressure was 7.7% and 10.3% in the McCoy group, respectively (p<0.001) [12]. However, in our study, the hemodynamic response was compared between the Miller and McCoy groups, where the same significant changes were observed in hemodynamic parameters (p<0.01).

Limitations

The study has some limitations, like a small sample size. Since laryngoscopy and endotracheal intubation are very common procedures performed under general anesthesia, the number of cases may be increased even more. Moreover, if the catecholamine levels could have been evaluated in the present study, then it would have certainly provided more strength to the study. Due to the costs involved in the above-mentioned biochemical marker, the catecholamine levels could not be assessed.

## Conclusions

The hemodynamic response to laryngoscopy and intubation, as shown by the increase in heart rate, systolic, diastolic and mean arterial pressure, was significantly lower with the McCoy blade as compared to the Miller blade. Although the Miller blade has its own distinct advantages in that it needs less force exertion, head extension, and minimal cervical spine movement, the flexible tip of the McCoy blade provides minimal alterations in the hemodynamic responses to laryngoscopy.
